# Impact of do‐it‐yourself air cleaner design on the reduction of simulated wildfire smoke in a controlled chamber environment

**DOI:** 10.1111/ina.13163

**Published:** 2022-11-25

**Authors:** Amara L. Holder, Hannah S. Halliday, Larry Virtaranta

**Affiliations:** ^1^ Office of Research and Development U.S. Environmental Protection Agency Research Triangle Park North Carolina USA; ^2^ Jacobs Technology International Research Triangle Park North Carolina USA

**Keywords:** air filtration, air purifier, filter fan, fine particulate matter, indoor air quality, wildfire smoke

## Abstract

During wildfire smoke events public health agencies release advisories to stay indoors, close doors and windows, and operate a portable air cleaner (PAC). The do‐it‐yourself (DIY) air cleaner consisting of a box fan and a furnace filter is a widely used low‐cost alternative to commercial PACs because of its increased accessibility. In this study, we evaluate the clean air delivery rate (CADR) of different DIY air cleaner designs for reducing simulated wildfire smoke and identify operating parameters that may impact their performance and use. The simplest formulation of a DIY air cleaner (box fan with taped on minimum effectiveness reporting value – [MERV] 13 furnace filter) had a CADR of 111.2 ± 1.3 ft^3^/min (CFM). Increasing the fan flow by changing the fan type, increasing the fan setting, or reducing the pressure drop across the filtering surface increased the CADR. Large increases in CADR could be obtained by using a shroud (40%), using a 4″ thick filter (123%) using two filters in a wedge shape (137%), or using four filters in a Corsi‐Rosenthal (CR) box design (261%). The CADR was greatly reduced with filters heavily loaded with smoke, pointing to the need for frequent filter changes during smoke events.

**Practical Implications**

Every year wildfires degrade air quality throughout large parts of the United States exposing millions of people to poor air quality. PACs are an effective way of reducing indoor fine particulate matter (PM_2.5_) but often quickly sell out during smoke events and have a cost barrier. Low‐cost DIY air cleaners can be built out of inexpensive materials that are more accessible during smoke events than commercial air cleaners. This study demonstrates that DIY air cleaners are an effective approach to achieve cleaner indoor air, despite higher noise levels and power requirements than a similar capacity commercial air cleaner. However, DIY air cleaner designs with a cardboard shroud and multiple filters increase the cost effectiveness of DIY air cleaners making them more effective than higher‐priced commercial units. The DIY air cleaner was almost completely ineffective with dirty filters, highlighting the need for frequent filter replacement during smoke events.

## 1 INTRODUCTION

For many years wildfires have been the single largest source of fine particulate matter (PM_2.5_) to the atmosphere in the U.S.,^1^ leading to an increase in average PM_2.5_ concentrations in the western part of the country despite long‐term trends of decreasing anthropogenic pollution.^2^ Wildfires are also a major source of ambient air pollution globally and are one of the largest sources of PM_2.5_ in some parts of the world (e.g., sub‐Saharan Africa and some regions in Asia, North America, South America, and Europe).^3^, ^4^ Smoke from wildfires can lead to localized extreme air quality impacts but can also travel hundreds of miles and impact large population centers downwind.^5^, ^6^ Smoke concentrations can remain elevated for days to weeks leading to long exposure durations in affected communities.^5^

During wildfire smoke events, standard public health guidance is to go indoors, close doors and windows, and operate an air cleaner to reduce smoke exposures.^7^ There is some evidence that moving indoors can reduce exposure to smoke,^8^ but smoke can readily infiltrate indoors and lead to unhealthy concentrations.^9^ Additionally, in temperate climates where central air systems are less common, closing doors and windows to keep out smoke traps in heat, leading to a tradeoff between exposure to excessive heat or smoke.

Using a portable air cleaner (PAC) has been identified as an effective method to reduce indoor PM_2.5_ concentrations and reduce health effects.^10^, ^11^ However, some PACs can be expensive and often increase in price with air cleaning capacity, making them inaccessible to some socioeconomic groups leading to health disparities. Additionally, PACs may be inaccessible due to limited market availability during smoke events.^12^ The recurrent need for cleaner air indoors during smoke events and limited accessibility for all impacted communities has led to the widespread use of do‐it‐yourself (DIY) air cleaners, also called filter fans. Numerous examples and guides exist online with the most common design consisting of a 20″ box fan with a single furnace filter attached to the fan inlet. Safety concerns on using a box fan with the filter in an aftermarket modification prompted a safety evaluation by Underwriters Laboratory. They demonstrated that even under a worst‐case scenario, both fan sides completely obstructed, resulted in no unsafe temperatures. The low cost and evidence of safe operation may lead to more widespread DIY air cleaner use.^13^ Some local air quality agencies have begun recommending that members of their communities make DIY air cleaners to improve indoor air quality during smoke events, with some organizations distributing DIY air cleaners^14^ or providing the raw materials to facilitate their use during smoke events (e.g., Puget Sound Clean Air Agency).

DIY air cleaners have also risen in popularity as a low‐cost method to reduce risk of COVID‐19 transmission indoors,^15^ with further design improvements to increase their cleaning capacity. These designs optimize the filtration surface area by using 4 or 5 filters attached to a single box fan (Corsi‐Rosenthal Box, CR Box) increasing the filtered air flow.^16^ Although there are multiple designs and guides for DIY air cleaners, there is very little information on their ability to deliver clean air and how their effectiveness may compare to more costly commercial PACs.

PAC effectiveness can be evaluated by measuring the single pass removal efficiency (fraction of particles removed from the inlet concentration) or by measuring the particle concentration decay due to the device.^17^ Effectiveness is often reported as the clean air delivery rate (CADR) quantified in terms of clean air flow in ft^3^/min (CFM) and sometimes in m^3^/hr. Initial DIY air cleaner evaluations have focused on COVID risk reduction and have found them to be very effective at reducing particle concentrations. Reported CADRs for DIY air cleaners range from 49 CFM^18^ to 825 CFM,^19^ depending upon the fan/filter features and particle characteristics. CADR increased with increasing fan speed,^18^, ^19^, ^20^ increasing filter thickness,^20^ increasing filter rating (i.e., minimum effective reporting value – MERV),^20^ increasing number of filters used^18^, ^20^ and increasing particle size.^21^

However, there is large variability in the reported CADR for the basic design of a single MERV 13 filter with a box fan operated at high‐speeds: 80,^18^ 300^20^ and 330 CFM.^22^ Similarly, a large range of CADRs (168–825 CFM) have been observed for the 5‐filter CR box.^18^, ^19^, ^20^, ^21^ The range in reported CADRs may be impacted by specific design features used in each study (e.g., filter or fan manufacturer), but also by the evaluation method. For example, Pistochini and McMurry^18^ estimated CADR by measuring flow through the fan and applying the rated efficiency of the filter, Srikrishna^20^ estimated the single pass efficiency by measuring the particle number concentration with the fan on and off, and others measured decay rates with salt^19^ or incense.^21^ These differences across studies make it difficult to compare DIY air cleaner performance across designs and in relation to commercial air cleaners, which are often characterized by a certified CADR following the measurement protocol developed by the Association of Home Appliance Manufacturers (AHAM).^23^

There has not yet been a systematic evaluation of the impact of DIY air cleaner design on effectiveness and usability features, like cost, energy use, and noise levels, which can be major barriers to PAC use. The objective of this study is to measure the CADR of a variety of different DIY air cleaner designs in a method comparable to the AHAM certification testing and identify the impacts of some common use scenarios (e.g., dirty filters). This information is valuable for identifying tradeoffs between DIY and lower‐cost commercial PACs and developing optimal guidelines for their use during wildfire smoke events.

## 2 EXPERIMENTAL METHODS

A chamber the size of a small room (29.3 m^3^, diagram and picture in the Figure S1) was used to simulate the conditions in the AHAM certification test for portable air cleaners.^23^ The chamber and its operation were previously described in detail by Liu et al.^24^ and are briefly described here. The chamber is climate controlled to maintain the temperature at 22.7 ± 0.8°C and provide clean air (filtered and passed through a carbon adsorber) at a low air exchange rate (0.7 ± 0.09 h^−1^). The relative humidity (RH) of the supply air was controlled with a dehumidification and humidification system that kept the chamber at 36.6% ± 8.9%.

The chamber air was continuously monitored for CO_2_ (LiCor820, LiCor BiosciencesNE), CO (U300, Teledyne APICA), and total hydrocarbon (THC, Flame Ionizer Detector CAI600, CAI Orange) concentrations. PM_2.5_ was measured continuously with a DustTrak (DRX, TSI Inc, Shoreview, MN). PM was collected on the DustTrak internal Teflon filter and amassed across multiple experiments. Filters were equilibrated at 25°C and 35% relative humidity for at least 24 h before being weighed on a microbalance (Mettler Toledo XPR2U). The DustTrak continuous PM_2.5_ concentrations were then corrected using the filter‐based correction factor of 0.2, similar to the 0.25 measured by Delp and Singer^25^ for wildfire smoke.

The power draw from the fan was measured with a continuous plug load power logger (U120‐018, Onset), which measures the active power at 10 second time resolution and an accuracy of 0.5 W (calibrated by the manufacturer in accordance with ANSI C12.20). The sound level (dBA) in the chamber was measured with a sound meter (Digi‐Sense 202 050–29 sound meter, Cole‐Parmer) placed 2 ft from the base of the fan outlet. The sound meter measures dBA at a 1 second time resolution and an accuracy of 1.4 dB (conforming to IEC61672‐1 Class 2 standards). The device sound level over the background sound level (from the chamber ventilation system and sampling equipment) was calculated following the same procedure used by Singer and Delp^26^ to evaluate in‐use kitchen exhaust hoods:

(1) AC=10log1010T10−10CH10,

where AC is the sound level attributed to the air cleaner, *CH* is the background chamber sound level (with the fan off), *T* is the total sound level with the air cleaner on. Measurements were repeated in an office environment (3.0 × 3.1 × 2.7 m, 25.1 m^3^) with lower background noise. The air flow through the system was estimated using a balanced‐pressure flow hood method described by Singer et al.^27^ with a variable speed fan (Duct Blaster, The Energy Conservatory). Measured flow rates exhibited high variability so only qualitative descriptions of the flow through the fan are reported here.

A smoke generation system was developed to inject a mixture of flaming and smoldering PM_2.5_ from burning pine needles into the chamber. The system consists of a 1″ diameter tube furnace maintained at approximately 800°C. Approximately 0.34 g (high smoke concentration) or 0.12 mg (low smoke concentration) were put in a ceramic boat and inserted into the preheated furnace. Combustion air was provided at 1 L/min. An eductor (TD110LSS, Air‐Vac) operating with 20 L/min (high smoke concentration) or 10 L/min (low smoke concentration) of nitrogen or air was used to dilute and inject the tube furnace effluent into the chamber. Two small fans inside the chamber were used to mix the air before the air cleaner was turned on.

The CADR for each design/condition was measured using the AHAM test protocol AC‐1^23^ with at least three replicates for each condition. We deviated from the test protocol, which is carried out in a sealed chamber with no external air flowing in, by supplying a continuous low flow to the chamber as makeup air for instrument samples. The natural PM_2.5_ decay of the open chamber is larger than the closed room described in AC‐1^23^ and is accounted for by subtracting the natural PM_2.5_ decay rate from the decay rate when the air cleaner is operating. Natural decay measurements were made periodically throughout the testing with the target PM_2.5_ concentration to ensure representative values were used in the CADR calculation.

The basic DIY air cleaner was modeled after those provided by air quality agencies (i.e., the Puget Sound Clean Air Agency) to their communities or through popular online construction videos or blogs. We used one 20″ 3‐speed box fan model A (83 W, 1820 CFM) for most experiments as it is easily obtained at most hardware stores. The impact of fan type was determined by using two other box fans, model B nominally 100 W and model C nominally 75 W. These two fans are typical of commercially available models with flows of approximately 2000 CFM for the 100 W fan and 1800 CFM for the 75 W fan. Fan technical specifications are listed in Table S1. All fans had three speed settings designated here as ‘low’, ‘medium’, and ‘high’ (fan label of ‘1’, ‘2’, and ‘3’), corresponding to increasing flow and increasing power draw. In most experiments 1″ thick MERV 13 furnace filters with electrostatic media (electret filters) from a single manufacturer were used. Additionally, 4” MERV 13 filters and 1” MERV 11 filters from the same manufacturer were used to evaluate the impact of filter type. The DIY designs that were evaluated are summarized in Table 1 and shown in Figure S2.

**TABLE 1:** DIY air cleaner fan, filter, and designs evaluated in this study

Test name	Filter type	Filter loading	DIY design	Fan type/Fan speed setting
MERV13‐1″ (baseline)	MERV 13 1″	Clean	Filter taped to inlet	Fan A/High, medium, low
MERV11‐1″	MERV 11 1″	Clean	Filter taped to inlet	Fan A/High
MERV 13‐4″	MERV 13 4″	Clean	Filter taped to inlet	Fan A/High
Bungee	MERV 13 1″	Clean	Filter bungeed to inlet	Fan A/High
Smoke	MERV 13 1″	Smoke loaded	Filter taped to inlet	Fan A/High
Dust	MERV 13 1″	Dust loaded	Filter taped to inlet	Fan A/High
Fan B	MERV 13 1″	Clean	Filter taped to inlet	Fan B/High
Fan C	MERV 13 1″	Clean	Filter taped to inlet	Fan C/High
Outlet	MERV 13 1″	Clean	Filter taped to outlet	Fan A/High
Corner	MERV 13 1″	Clean	Filter taped to inlet, placed in corner of room	Fan A/High
Shroud	MERV 13 1″	Clean	Cardboard shroud	Fan A/High
Wedge	MERV 13 1″	Clean	2 − filter wedge + shroud	Fan A/High
CR Box	MERV 13 1″	Clean	4 − filter CR Box + shroud	Fan A/High

The filter (or filters) was attached to the box fan using duct tape or bungee cords and pieces of cardboard. A shroud consisting of a cardboard cutout that blocked portions of the fan outlet past the fan blade tips (Figure S2B) was used on the single filter design, 2‐filter wedge design, and 4‐filter CR box designs. The shroud prevents air recirculation in the fan increasing the total flow through the fan.^28^ For most conditions, the air cleaner was placed in the center of the chamber near the location of the smoke injection. For one set of tests, the air cleaner was placed in the corner of the room 20 inches from the wall to the center of the fan.

The impact of filter loading was determined by pre‐loading a MERV 13 1″ filter with ASHRAE test dust or simulated wildfire smoke. Dust loaded filters were generated by a commercial lab (Blue Heaven Technologies) using the ASHRAE 52.2 dust loading procedure^29^ to load filters with 53 ± 4 g of test dust (2 in H_2_O pressure drop). Smoke loaded filters were generated in the EPA Open Burn Test Facility.^30^ The facility was filled with smoke from small batches of pine straw and three air cleaners were operated simultaneously to reduce the smoke concentrations and load the filters. The process was repeated until the air cleaners ceased to reduce smoke concentrations in the chamber. The amount of smoke deposited was determined from a pre‐ and post‐weigh of the filters on a balance (Scout, Ohaus). Additionally, the pressure drop across the smoke loaded filters was measured by a commercial laboratory (E‐Spin Technologies).

A lower‐cost, small commercial air cleaner with an AHAM CADR of 100 (CFM) for tobacco smoke was tested to compare the PM_2.5_ removal effectiveness, sound, and cost of operation to the different DIY air cleaner designs. The statistical significance of comparisons between different air cleaners and designs was determined using a two sample T‐test at a significance level of α = 0.05.

The material cost of the air cleaner was estimated from current prices on major online retailers (e.g., Amazon) and retailers with an online presence and physical locations in many rural areas (e.g., Ace Hardware). The median price of the limited survey done in April of 2022 was used to calculate the price per unit of each material needed to construct the air cleaner (Table S2). The operating cost was estimated by the power consumption for each air cleaner assuming a rate of 23.58 cents/kWhr for March 2022 in California (EIA 2022 https://www.eia.gov/electricity/data/browser/#/topic/). This electricity price was on the upper end of retail prices in the U. S. at the time and therefore provides an upper estimate of the operational cost of the air cleaner.

## 3 RESULTS AND DISCUSSION

### 3.1 General observations

PM_2.5_ concentrations were moderately repeatable within the chamber for both high and low concentration conditions, with approximately 30% variation in the PM_2.5_ concentration across replicate tests. PM_2.5_ concentrations were reduced to baseline levels between tests by the air cleaner, but the gas phase species (e.g., CO or THC) slowly increased in the chamber as multiple tests were run each day. The natural decay rate in the chamber was measured routinely throughout the study and was 0.012 ± 0.001 min^−1^ (*n* = 32). The natural decay varied by 8.3% over the duration of the study due to changes in the instrument sample flow, as the number and type of instruments pulling samples from the chamber varied.

### 3.2 Factors impacting DIY performance

**FIGURE 1:**
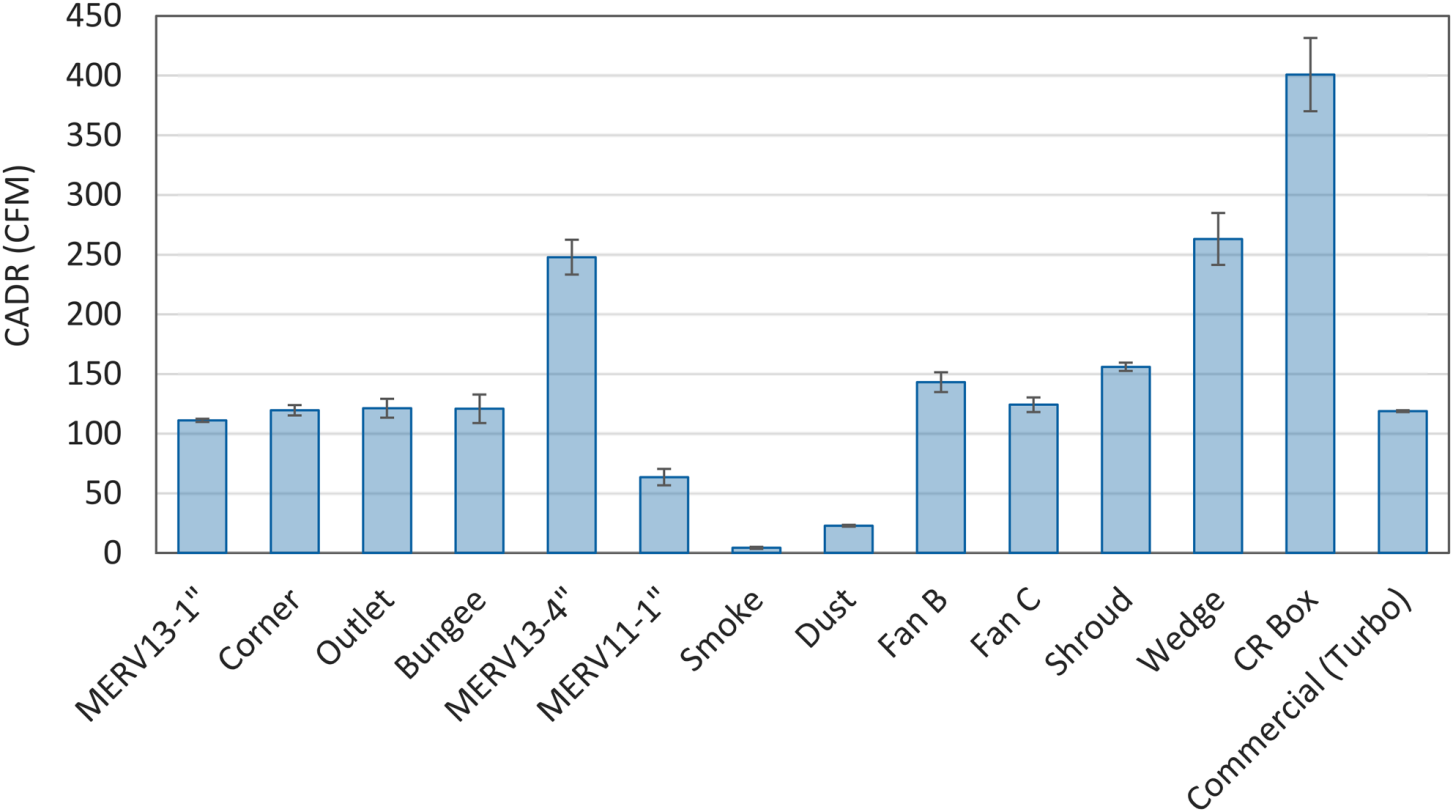
CADR for the DIY air cleaner conditions listed in Table 1.

Unlike commercial air cleaners, DIY air cleaners can be constructed and used in different ways that may impact CADR. We investigated a range of PM_2.5_ concentrations, design features, and filter loading conditions to better understand what factors were most important in determining the CADR (Figure 1). The single MERV 13–1″ filter taped to a box fan was used as the baseline scenario to which all other conditions were compared. This baseline design is the simplest to construct, extensively publicized, and, in some cases, distributed by air quality agencies. It is therefore likely to be most widely used for reducing wildfire smoke. The baseline DIY design had CADR from 79.7–111.2, depending on fan speed (Table 2), which is comparable to a small capacity commercial air cleaner suitable for small rooms. We also included the CR box in the evaluation as it has increased in popularity as a low‐cost method to reduce COVID transmission risk indoors^31^ and has been evaluated in several other studies.^18^, ^19^, ^20^, ^21^

**TABLE 2:** CADR for different fan configurations and test conditions at high fan setting, unless noted

Condition	CADR (CFM)	Device noise level (dbA)	Power consumption (W)	Initial cost ($)^a^	Weekly operating cost ($)^b^
Baseline Low fan speed	79.7 ± 3.1	35	58.8 ± 0.2	45.38	0.78
Baseline Medium fan speed	96.9 ± 2.1	–	67.5 ± 0.6	45.38	0.89
Baseline High fan speed	111.2 ± 1.3	55	77.1 ± 0.8	45.38	1.02
Corner	119.6 ± 4.4	–	78.4 ± 0.6	45.38	1.04
Smoke	4.31 ± 0.9	–	76.1 ± 0.6	45.38	1.00
Dust	22.9 ± 0.8	–	77.8 ± 0.3	45.38	1.02
Bungee	120.9 ± 11.9	63	77.8 ± 0.2	42.78	1.03
Outlet	121.3 ± 7.9	64	78.6 ± 0.3	45.38	1.04
MERV11‐1″	63.6 ± 6.8	63	78.3 ± 0.6	43.65	1.03
MERV13‐4″	247.9 ± 14.6	63	77.7 ± 0.3	72.81	1.03
Fan B	143.2 ± 8.2	75	104.8 ± 0.7	70.38	1.38
Fan C	124.3 ± 6.1	62	71.8 ± 0.4	39.38	0.95
Shroud	156.1 ± 3.6	62	77.6 ± 0.2	45.38	1.02
Wedge	263.1 ± 21.8	61	76.1 ± 0.4	54.78	1.01
CR box	400.9 ± 30.7	55	76.0 ± 1.1	73.59	1.00
Commercial Turbo fan speed	118.9 ± 0.7	51	41.1 ± 0.2	123.05	0.54

^a^ Prices obtained from national retailers in April 2022, filter unit price derived from a pack of 6.

^b^ Prices estimated with the air cleaner being used for 8 h per day 7 days a week at an electricity cost of 23.58 ¢/kWh average price for CA in March 2022.^39^ This does not include price of replacement filters.

### 3.3 Initial PM_2_ _.5_ concentration

**FIGURE 2:**
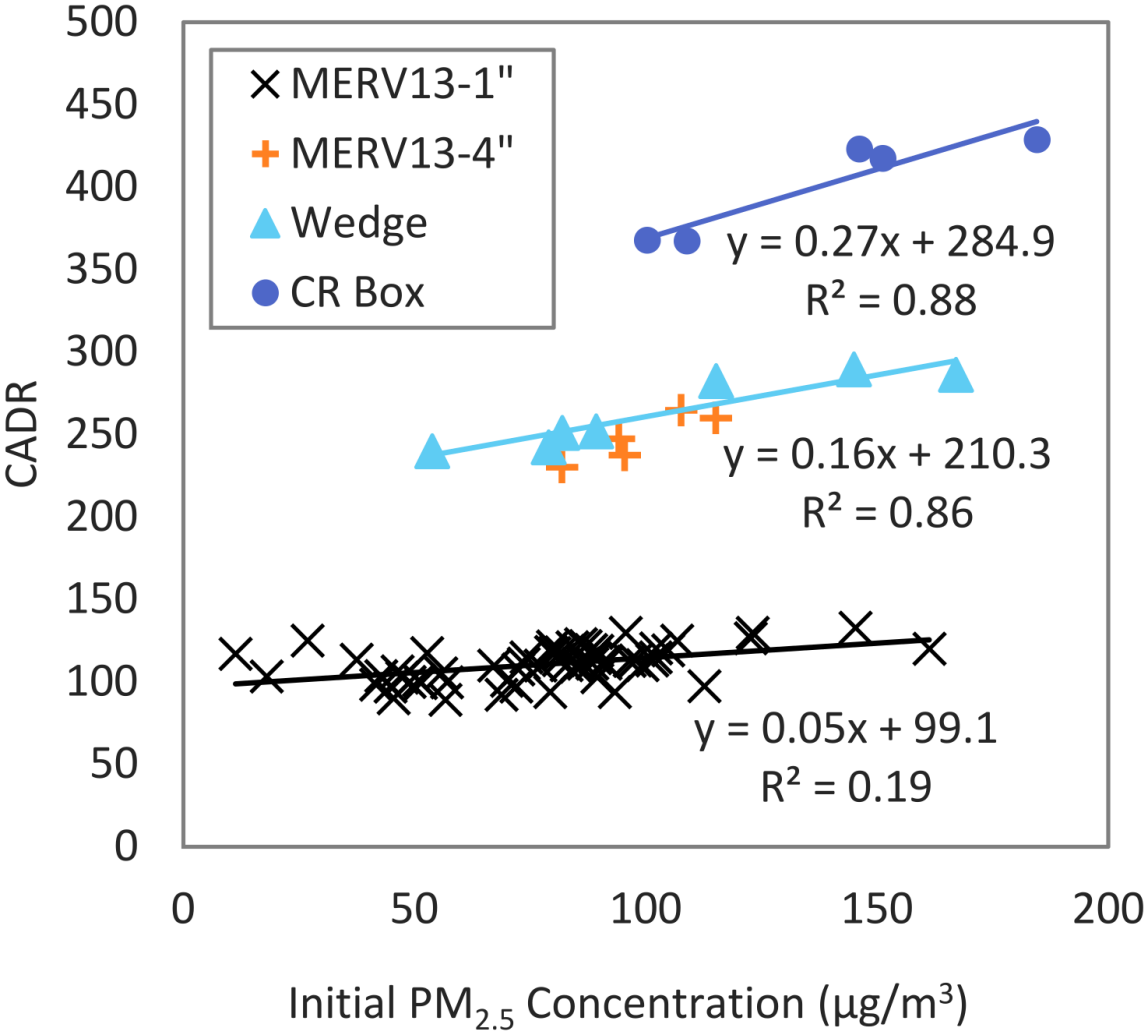
Variation of the CADR with the initial PM_2.5_ concentration in the chamber for different DIY air cleaner designs. Data for MERV13‐4″ design overlaid to show similarity with Wedge design, but not included in linear regression.

The CADR tended to increase with increasing initial PM_2.5_ concentration, but only for designs with high CADR (Figure 2). The baseline scenario (MERV 13 1″ filter, attached by either tape or bungee cord) exhibited minimal variation in CADR across a wide range of initial PM_2.5_ concentrations (11 to 161 μg/m^3^), representing good, moderate, unhealthy, and very unhealthy air quality conditions using the U.S. Air Quality Index (AQI).* However, the CADR of the wedge design was strongly correlated with initial PM_2.5_ concentration (*r* ^2^ = 0.86 for PM_2.5_ = 54–167 μg/m^3^), as was the CR box (*r* ^2^ = 0.87 for PM_2.5_ = 100–185 μg/m^3^). The relationship between CADR and initial concentration is linear, and the slope of this relationship increases with the increasing CADR of the more effective DIY designs. The variation in the slope with CADR was not dependent on DIY design as the 4” MERV 13 CADR range overlapped the wedge CADR, despite having a different number of filter faces. CADR is not expected to change with concentration,^17^ but this may reflect a shift to larger particle sizes at higher concentrations, which may be removed more efficiently with MERV 13 filters than the smaller particles at lower concentrations. The particle size was not measured but a proxy through a size ratio (PM_2.5_/PM_10_) derived from PurpleAir sensor measurements suggests that particle size increases with PM_2.5_ concentration (Figure S3). However, the DustTrak reported nearly identical concentrations for PM_1_, PM_2.5_, PM_4_, and PM_10_, suggesting that all the particles were in the PM_1_ size fraction. Although both instruments use light scattering to measure and predict size fractions, their design and calculation algorithm clearly differ, and additional sizing measurements would be needed to determine how the size may vary with concentration in the chamber.

### 3.4 DIY air cleaner operation: Fan setting and fan placement

The CADR was strongly dependent on fan setting with the highest fan setting providing the greatest CADR but also the largest noise levels and highest power draw (Table 2). Increasing the fan setting led to a statistically significant and linear increase in CADR. Location of the DIY air cleaner in the room did not impact the CADR, which was approximately constant for the air cleaner in the center and in the corner facing the room or facing the corner. The minimal impact of the location of the air cleaner within the chamber is perhaps not surprising given that the fan was operated at high speed in a single well‐mixed room with no obstructions. The DIY air cleaner has a moderate CADR but can deliver high flow rates (nominally 1800 CFM), which likely contributes to mixing within the chamber, lessening the impact of air cleaner location.^32^ A similar study by Küpper et al.^33^ in a single room office also found minimal impact of the air cleaner location, except for the case with the air cleaner was underneath a table that obstructed flow.

### 3.5 DIY air cleaner design: Fan model, MERV rating, and filter configuration

The fan model had a small but significant impact on the CADR. The higher wattage and more expensive fan, fan B, ~$55) had a 29% higher CADR, but the higher power draw also translates to a higher operating cost. The less expensive fan, fan C (~$24) had a CADR that was 12% higher than the baseline fan, fan A (~$30), demonstrating that the cost of the fan is not the sole determinant of potential CADR in DIY air cleaners. Fan costs may be impacted by other design features or branding that do not impact air flow and may have no impact on potential CADR.

The MERV rating of the filter strongly impacted the CADR with a 43% reduction in CADR by switching from a MERV 13 to a MERV 11, with almost no change in the power draw or the device noise. Although MERV 11 filters can sometimes be marketed as effective at removing smoke, the specifications for the MERV 11 rating require only 20% efficiency in removing particles smaller than 1 μm.^29^ Wildfire smoke generally has a geometric mean diameter approximately 0.3 μm,^5^ and most smoke particles fall into the range where MERV 11 is least effective, translating to a lower CADR.

A thicker filter (4″ compared to 1″) with the same MERV 13 rating increased the CADR by 123%. The thicker pleated surface provides a larger surface area, reducing the pressure drop, and resulting in higher flows through the fan.

The DIY air cleaner design had a large impact on CADR and was the most effective approach to increase the CADR delivered from a single fan without adjusting fan setting or changing power requirements. The addition of a cardboard shroud (Figure S2B) increased the CADR by 40% without any change in the cost or physical footprint. Increasing the number of filters used in the design increased the cost of the raw materials to construct the air cleaner and the size of the air cleaner but was also very effective at increasing the CADR. The CADR for the wedge design was increased by 137% and for the CR box by 261% over the baseline scenario. These simple design modifications change the capacity of the DIY air cleaner making it suitable for larger rooms, more representative of the living spaces in the home and comparable to commonly marketed and highly recommended air cleaners.

### 3.6 Filter condition

The filter condition had a strong impact on DIY air cleaner effectiveness. The DIY air cleaner was almost completely ineffective when used with dirty filters. The CADR for the smoke loaded filter was 4.3 ± 0.9 CFM and repeated with a second smoke loaded filter (CADR = 9.7 CFM) to confirm the poor performance. The smoke loaded filters had only 10.0 ± 1.1 mg of smoke deposited and, despite being only a small mass of smoke, resulted in dark filters with a distinct smoke smell. We qualitatively assessed the air flow through the fan and found that it was largely unaffected by the smoke loaded filters. Additionally, the pressure drop across the filter was only slightly higher (0.29 ± 0.01 in H_2_O at 819 CFM) than the clean filter (0.27 ± 0.01 in of H_2_O at 819 CFM). Therefore, it is unlikely that the low CADR is due to the quantity of smoke deposited on the filter. It is likely that the low CADR for the smoke loaded filters is due to the electrostatic filters that were used in this study. Fresh smoke particles can carry charge^34^ and when deposited onto the filter build up a charge on the surface that serves to repel charged smoke aerosol. This loss of effectiveness has been observed with electrostatic filters loaded with cigarette smoke^35^ and the effectiveness could be restored by discharging the filter using isopropyl alcohol.^36^ The loss of effectiveness through charge buildup also explains why the flow was not greatly reduced but the filter was still ineffective at reducing PM_2.5_ concentrations in the chamber.

The dust loaded filters had a much higher pressure drop than the clean filters (2 vs. 0.27 in H_2_O at 819 CFM) and exhibited much lower CADR at 22.9 ± 0.8 CFM. The dust loaded filters have five times higher CADR compared to the smoke loaded filters, despite having three orders of magnitude greater mass loading. This further suggests that the unique characteristics of smoke particles impact the CADR and highlight the importance of using a realistic test aerosol to estimate air cleaner effectiveness in different use scenarios. This large difference in the loading capacity between smoke and dust will likely vary by filter and may end being similar with filters that are not electrostatic like those used here. Further study with other filter types is needed to confirm this. The practical implication of this difference between smoke and dust loading is that the DIY air cleaner may remain effective for dust for longer times and higher dust concentrations, than for wildfire smoke. More frequent filter changes may be necessary to maintain effectiveness for wildfire smoke events.

The filter type, filter condition, or DIY design had no significant effect on the noise level or the power requirements. Only the CR box slightly decreased the noise levels at the highest fan setting, however this was primarily due to the direction of the fan output with respect to the microphone. For example, the fan output in the CR box faces upward while the microphone was placed on the floor in front of the DIY air cleaner for all tests. The fan type and fan setting had the largest impact on noise levels and power draw. Fan B had the highest power draw and noise levels of all three fans tested and commensurately had the highest CADR. Fans A and C had similar noise levels and power draws, but fan C had a 12% higher CADR.

### 3.7 Comparison with other studies

**FIGURE 3:**
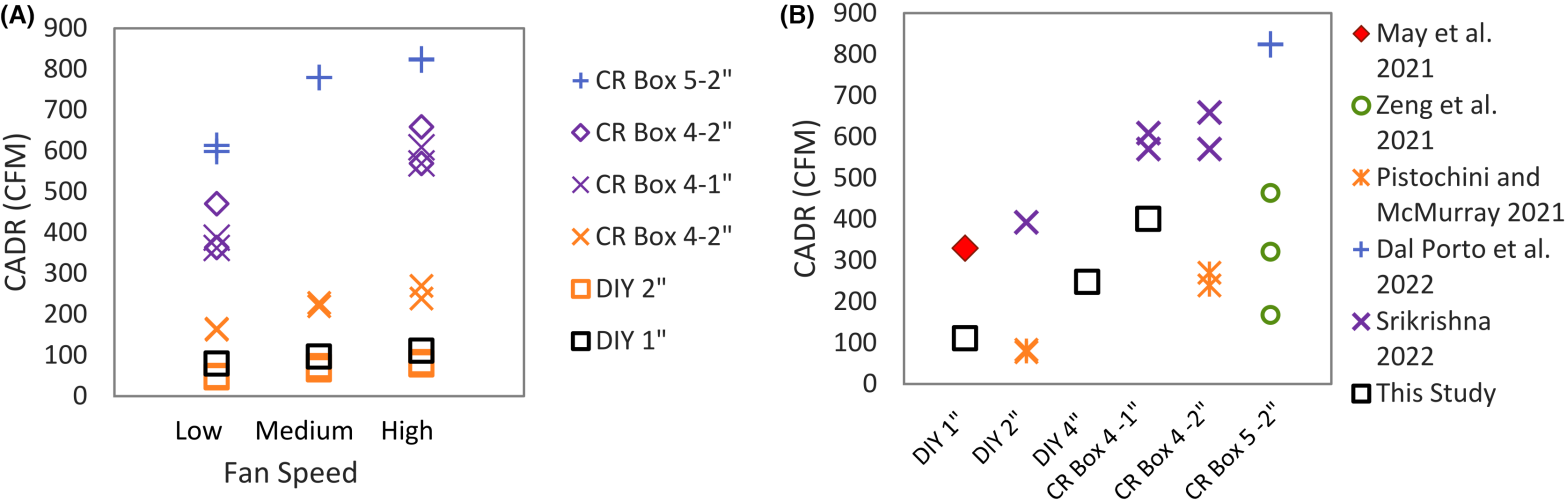
Comparison of literature reported values of (A) CADR versus fan setting using different DIY designs, blue crosses from Dal Porto et al. 2021, purple diamonds and x's from Srikirshna 2022, and orange x's and squares from Pistochini and McMurray 2021 and (B) CADR versus DIY design all at the highest fan setting compared to current study findings noted with black squares.

The CADR measured in this study are compared to other studies evaluating DIY air cleaners in Figure 3. There is a consistent increase in CADR with increasing fan settings across studies (Figure 3A), but the CADR for a single fan setting varies greatly, in part due to different design aspects. This can be seen when comparing CADR for the highest fan setting for different designs and studies in Figure 3B. There is a clear trend of increasing CADR with increasing filter thickness and increasing number of filters used in the design, but large differences remain across studies examining the same configuration due to the CADR estimation method. The impact of test aerosol is seen by Zeng et al.^21^ that reported increasing CADR for a CR box with increasing incense particle size (0.09–1, 0.5–3, 5–11 μm) compared to Dal Porto et al.^19^ for the same CR box design with 0.5–5 μm nebulized salt particles. In addition to the particle composition and size range, the CADR estimation method impacts the measured value. For example, Srikrishna^20^ using a measured particle removal efficiency and flow reports consistently higher CADR than all other studies, and Pistochini and McMurry^18^ used a measured flow with the filter rated removal efficiency and reported consistently lower CADR than all other studies. These method dependent differences can amount to CADRs that vary by 1.5–3 times resulting in coefficient of variation of 20% (CR Box 4 – MERV 13 1″ filters) to 100% (DIY MERV 13 filters) across studies.

### 3.8 Implications

This study has shown that DIY air cleaners can reduce smoke concentrations indoors and is a viable approach to improve indoor air quality during smoke events and provides CADR that are comparable to lower‐cost commercial air cleaners sized for smaller rooms. Designs that minimized the pressure drop across the fan by increasing the filter surface area (e.g., use of one 4″ thick filter, 2 or 4 filter designs) were able to achieve the highest CADR without any increase (or decrease) in noise levels or power consumption. These designs led to an increased CADR that were comparable to more commonly marketed commercial air cleaners (approx. 300 CFM) that is more appropriate for larger living spaces.

The CR box was particularly effective in reducing PM_2.5_ concentrations with CADR nearly four times that of DIY air cleaner with a single MERV 13 1″ filter. In our small room‐sized chamber, the PM_2.5_ concentration was reduced from 80 μg/m^3^ to below 10 μg/m^3^ in approximately 6 min. However, this design is more complicated to construct and may take up a larger footprint, which may prevent widespread adoption of this design, despite the dramatically increased CADR from the baseline design of a single MERV 13 1″ filter.

**FIGURE 4:**
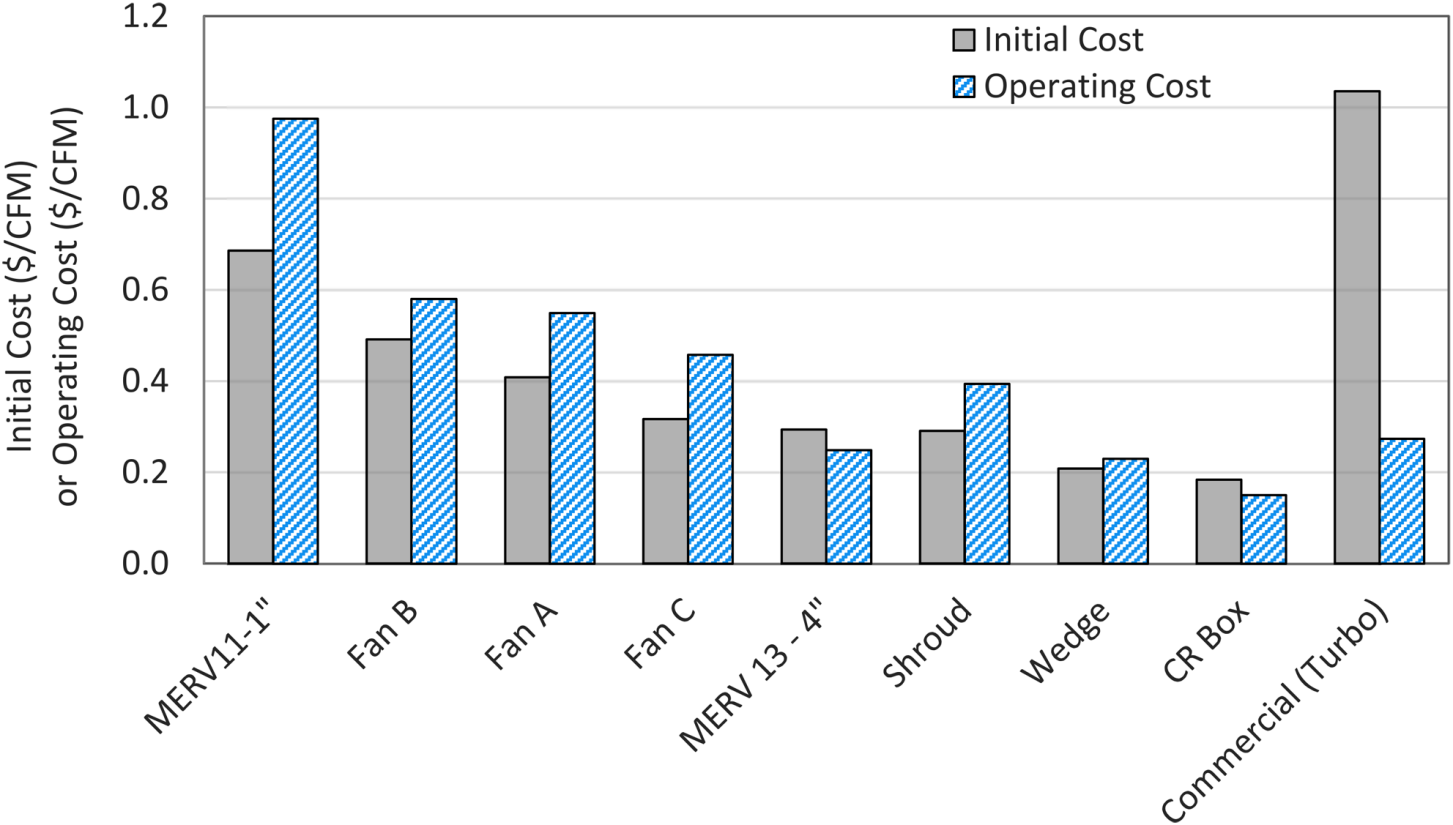
Initial cost of materials to build or purchase each air cleaner per unit CADR and estimated weekly operational cost assuming 8 h of use per day, 7 days a week and an electricity rate of 23.58 ¢/kWh.^39^

The cost to purchase commercial air cleaners can be a major barrier in low‐income communities and is one reason why DIY air cleaner designs have been so extensively publicized. Many people may already own a box fan and the air cleaner could be constructed with minimal cost by purchasing filters. Additionally, the lower material cost makes them attractive to air cleaner distribution programs run by local air quality agencies, community groups, or other non‐profit organizations. We compared the DIY air cleaner designs and a low‐cost commercial air cleaner by the cost per unit of clean air delivered to identify the lowest cost and effective design (Figure 4). Furthermore, we categorized the costs into: (a) the initial cost to construct or purchase the air cleaner and (b) an estimated operating cost, which is the price of the electricity used to power the air cleaner.

The price of different DIY designs did not vary as much as the CADR, so normalizing cost by CADR clearly separates the optimal designs from the less optimal versions. The CR box provides the lowest cost per unit clean air (0.18 $/CADR), followed by the wedge design (0.21 $/CADR), and single filter design using a 4” MERV 13 filter (0.29 $/CADR). All of these DIY designs had a much lower initial cost compared to the small commercial air cleaner. The estimated operating costs of these designs were also similar and lower than the commercial air cleaner, despite the lower energy consumption of the commercial air cleaner. Assuming the filter effectiveness degrades at a similar loading rate in all DIY air cleaner designs, replacement filter costs would be comparable since multi‐filter designs would last longer. However, further study on filter lifespan is needed to confirm this supposition.

Other factors, apart from CADR and cost, in achieving cleaner air indoors is the ease of use and the potential barriers to use. Potential barriers to the DIY air cleaner use are the louder noise levels, the larger space that it takes up, and the greater effort involved in constructing the air cleaner compared to the commercial air cleaner. Noise is a well‐known major barrier to air cleaner use as is the draft they may produce,^37^ but complexity of design is an additional consideration for DIY air cleaners since the user must also construct the cleaner. The CR box provides the best value (i.e., lowest initial and operating cost per CADR), but is more difficult to construct and takes up a larger amount of space. The single filter design using a MERV 13–4″ filter has a lower CADR but requires less space and is easier to construct. Despite a higher initial cost, the operating cost of the CR box may be lower since lower fan settings (and lower power draw) can effectively clean the same area as a single filter version.

### 3.9 Limitations and suggestions for further study

A limitation of this study is the small number of fans and filter types investigated. The combinations tested here are a small fraction of the potential DIY air cleaners that can be constructed given the multiple types of box fans and numerous types of filters that are commercially available. This study focused on the simplest designs that may be most likely to be constructed and so combinatorial designs were not tested. Further studies could look at designs that combine varying number of filters, filter rating, filters without electrostatic media, and filters from other manufacturers to identify the most cost‐effective design parameters. Although we showed that loaded filters caused a sharp decrease in the CADR, the heavy loading state used here is likely unrealistic for normal use and more study is needed to identify how the CADR changes over time as the filter load increases with more realistic smoke concentrations.

Testing in real environments is needed to truly measure the efficacy of DIY air cleaners, including the impact of noise levels and air flows on user behavior, which may greatly impact the achievable PM reductions. Moreover, the effectiveness of the DIY air cleaner in reducing symptoms is still needed to understand the benefits in addition to the costs associated with their routine use. Commercial air cleaners have been largely shown to be effective in reducing PM, but this has not always translated to improved health.^11^ However, given the sometimes high indoor PM concentrations observed near wildfires,^38^ DIY air cleaners may be very useful at improving indoor air quality conditions during smoke events.

## 4 CONCLUSIONS

This study has shown that DIY air cleaners can be a cost‐effective approach to reducing smoke concentrations. The CADR was minimally impacted by different approaches to attach the filter to the fan showing that DIY air cleaner performance may be largely independent from how the user constructs them. The most cost‐effective designs were those with multiple filters, but the use of a single 4” MERV13 filter was also highly effective and may be more suitable for small areas with minimal floor space that cannot accommodate the multi‐filter designs. More study is needed to identify the optimal time to change filters and the optimal filter to use, particularly for wildfire smoke as the electrostatic filters used here were found to lose their filtering ability at much lower levels of PM loading compared to dust. This highlights the need for frequent filter changes during smoke events when they may quickly load up with PM.

## AUTHOR CONTRIBUTIONS

**Amara Holder:** Conceptualization, Investigation, Formal Analysis, Writing – Original Draft Preparation. **Hannah Halliday:** Investigation, Writing – Review and Editing. **Larry Virtaranta:** Investigation, Writing – Review and Editing.

## CONFLICT OF INTEREST

The authors declare no conflict of interest.

## DISCLAIMER

The views expressed in this manuscript are those of the author(s) and do not necessarily represent the views or policies of the U.S. Environmental Protection Agency.

## Supporting information


Appendix S1:

## Data Availability

The data that support the findings of this study are openly available in EPA Environmental Dataset Gateway at https://edg.epa.gov.

## Endnote

*
https://www.airnow.gov/aqi/.
