# Supplementary material for: Impact of do‐it‐yourself air cleaner design on the reduction of simulated wildfire smoke in a controlled chamber environment
Source: Indoor Air. 2022 Nov 25;32(11):e13163. doi: 10.1111/ina.13163 (PMC9828579; doi:10.1111/ina.13163)
Supplement: Supplementary file 1 — Appendix S1: [file INA-32-0-s001.docx]

**Supplemental Information For: Particulate matter removal effectiveness from do-it-yourself air cleaners**

Amara L. Holder^1^, Hannah S. Halliday^1^, Larry Virtaranta^2^

^1^ Office of Research and Development, U.S. Environmental Protection Agency, Research Triangle Park, NC, USA

^2^ Jacobs Technology International, Research Triangle Park, NC, USA

**Number of Figures 2**

**Number of Tables 3**

| 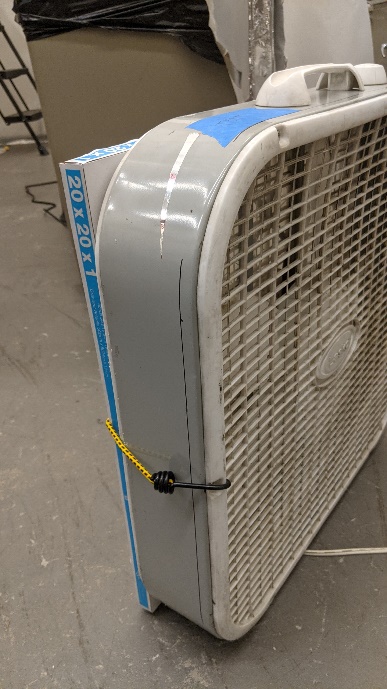(a) | (b)  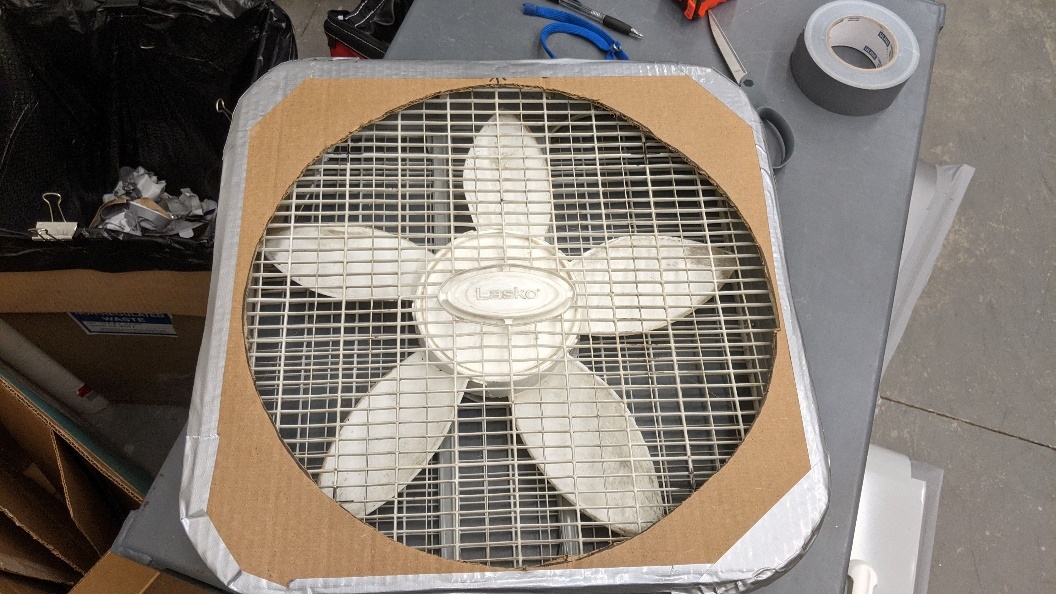 |
| --- | --- |
| 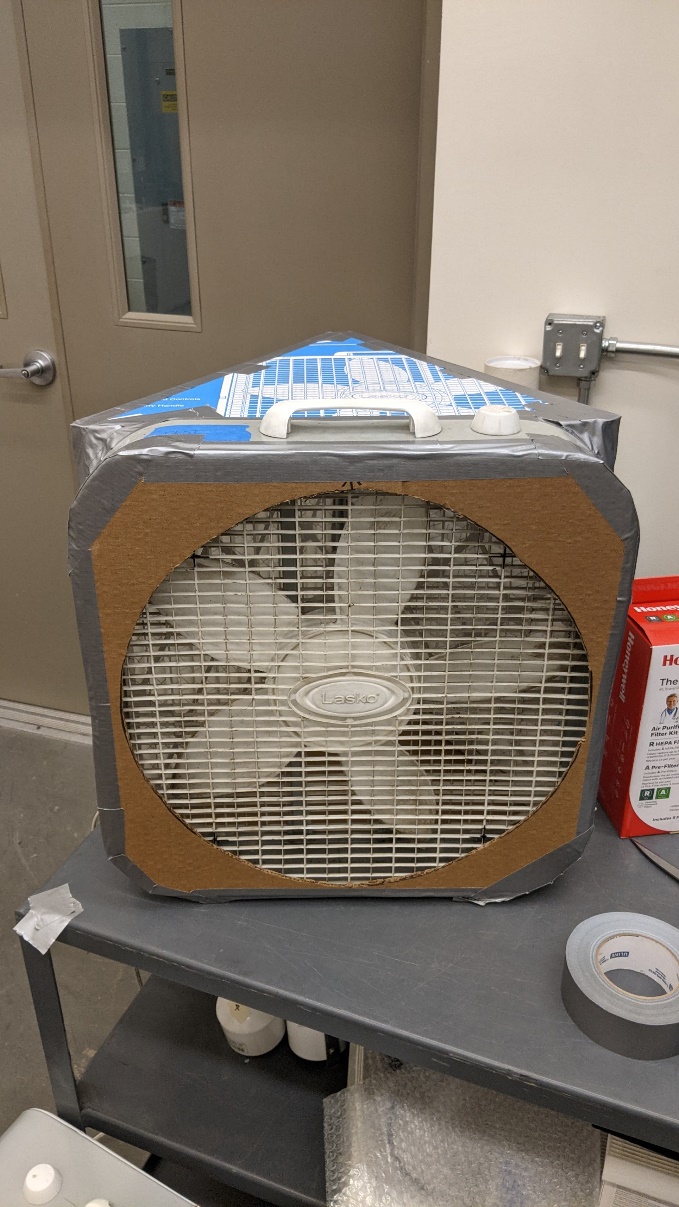(c) | 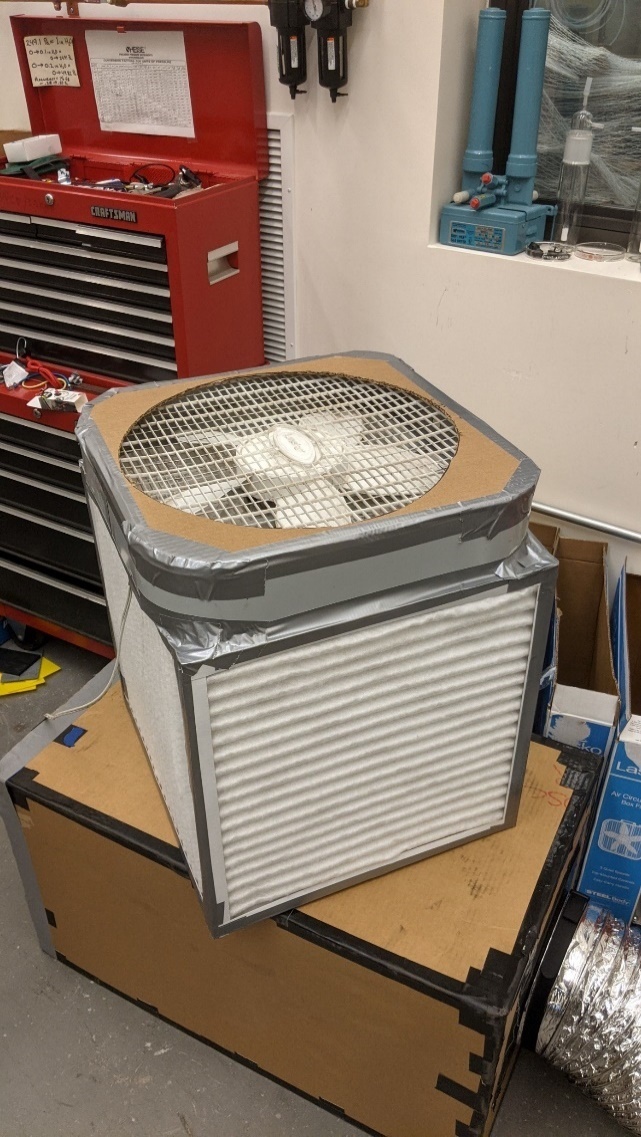(d) |

Figure SI-1 DIY air cleaner designs evaluated in this study (a) Fan A with MERV 13 1” filter bungeed to inlet, (b) Fan A with cardboard shroud, (c) Fan A with 2 MERV 13 1” fans in wedge design with cardboard shroud, (d) Fan A with 4 MERV 13 1” filters in CR box design with cardboard shroud

Figure SI-2 Ratio of PM_2.5_ concentration to PM_10_ concentration as measured by PurpleAir sensors during selected test conditions

Table SI-1 Fan/Air Cleaner specifications

| Specification | Fan A | Fan B | Fan C | Commercial |
| --- | --- | --- | --- | --- |
| Size | 20.5” x 4.25” x 21.75” | 20.5” x 6.375” x 23.625” | 21” x 4.2” x 21” | 14” x 13.5” x 8.9” |
| Weight | 7.25 lbs | 12.8 lbs | 6.4 lbs | 10.43 lbs |
| Number of speeds | 3 | 3 | 3 | 4 |
| Nominal Power Rating | 120V 60Hz 0.8A | 120V 60Hz 1.0A | 120V 60Hz 0.8A | 120V 60Hz 0.4A |
| Airflow | 1820 CFM | 2163 CFM | 1400 CFM |  |
| Safety listing | ETL – UL507 | ETL – UL507 | ETL – UL507 | ETL – UL507 |
| Smoke CADR |  |  |  | 100 |

Table SI-2 Price information for fan, raw materials, and electricity use for different DIY air cleaner configurations

| Air cleaner component | Price ($) | Date Recorded | Number of Units | Per Unit Price ($) |
| --- | --- | --- | --- | --- |
| Fan A | 29.99 | 4/3/2022 | 1 | 29.99 |
| Fan B | 54.99 | 4/3/2022 | 1 | 54.99 |
| Fan C | 23.99 | 4/3/2022 | 1 | 23.99 |
| Duct Tape (1.8" x 60') Roll | 5.99 | 4/3/2022 | 1 | 5.99 |
| Bungee (24" x 0.315") | 3.39 | 4/3/2022 | 1 | 3.39 |
| 20" MERV 13-1" (box of 6) | 56.41 | 4/3/2022 | 6 | 9.40 |
| 20" MERV 11-1" (box of 6) | 46.02 | 4/3/2022 | 6 | 7.67 |
| 20" MERV 13-4" (box of 1) | 36.83 | 4/3/2022 | 1 | 36.83 |
| Commercial air cleaner | 123.05 | 4/3/2022 | 1 | 123.05 |
| Commercial air cleaner replacement filter | 46.58 | 6/1/2021 | 1 | 23.29 |

Table SI-3 Test data for all air cleaners and configurations averaged across replicates.

| Condition | CADR (CFM) | Power (W) | Chamber Noise (dBA) | PM_2.5_ Concentration (µg/m^3^) | Temperature (C) | RH (%) |
| --- | --- | --- | --- | --- | --- | --- |
| MERV 13 - 1" low | 79.7 ± 3.1 | 58.8 | 58.97 | 108.1 | 21.4 | 41.6 |
| MERV 13 - 1" medium | 96.9 ± 2.1 | 67.5 | 64.59 | 118.5 | 22.4 | 36.2 |
| MERV 13 - 1" high | 111.2 ± 1.3 | 77.1 | 67.94 | 109.9 | 21.6 | 15.8 |
| MERV 13 - 1" corner | 119.6 ± 4.4 | 78.4 | 67.61 | 82.3 | 21.9 | 30.6 |
| MERV 13 –  On Front | 121.3 ± 7.9 | 78.6 | 64.48 | 98.2 | 22.7 | 33.9 |
| MERV 13 - bungee | 120.9 ± 11.9 | 77.8 | 67.28 | 109.7 | 23.5 | 33.9 |
| MERV 13 - 4" | 247.9 ± 14.6 | 77.7 | 67.47 | 98.8 | 23.4 | 34.5 |
| MERV 11 | 63.6 ± 6.8 | 78.3 | 66.76 | 83.2 | 23.2 | 35.7 |
| MERV 13 - Smoke Loaded | 4.3 ± 0.9 | 76.0 | 66.18 | 89.7 | 22.5 | 47.5 |
| MERV 13 –  Dust Loaded | 22.9 ± 0.8 | 77.1 | 66.80 | 112.7 | 23.2 | 38.2 |
| Fan B | 143.2 ± 8.2 | 104.8 | 77.71 | 98.3 | 23.5 | 38.7 |
| Fan C | 124.3 ± 6.1 | 71.8 | 65.67 | 98.0 | 23.3 | 37.3 |
| MERV 13 shroud | 156.1 ± 3.5 | 77.6 | 65.88 | 116.4 | 23.1 | 34.4 |
| Wedge | 263.1 ± 21.8 | 76.1 | 66.07 | 104.4 | 23.4 | 39.8 |
| CR Box | 400.9 ± 30.7 | 76.0 | 61.91 | 138.2 | 23.2 | 38.5 |
| Commercial (Turbo) | 118.9 ± 0.7 | 41.1 | 63.68 | 109.0 | - | - |
| Commercial (High) | 75.9 ± 0.6 | 26.9 | 53.91 | 121.7 | - | - |
